# Longitudinal RNA sequencing of the deep transcriptome during neurogenesis of cortical glutamatergic neurons from murine ESCs

**DOI:** 10.12688/f1000research.2-35.v1

**Published:** 2013-02-07

**Authors:** Kyle S Hubbard, Ian M Gut, Megan E Lyman, Patrick M McNutt

**Affiliations:** 1 United States Army, Medical Research Institute of Chemical Defense, MD, 21010, USA

## Abstract

Using paired-end RNA sequencing, we have quantified the deep transcriptional changes that occur during differentiation of murine embryonic stem cells into a highly enriched population of glutamatergic cortical neurons. These data provide a detailed and nuanced account of longitudinal changes in the transcriptome during neurogenesis and neuronal maturation, starting from mouse embryonic stem cells and progressing through neuroepithelial stem cell induction, radial glial cell formation, neurogenesis, neuronal maturation and cortical patterning. Understanding the transcriptional mechanisms underlying the differentiation of stem cells into mature, glutamatergic neurons of cortical identity has myriad applications, including the elucidation of mechanisms of cortical patterning; identification of neurogenic processes; modeling of disease states; detailing of the host cell response to neurotoxic stimuli; and determination of potential therapeutic targets. In future work we anticipate correlating changes in longitudinal gene expression to other cell parameters, including neuronal function as well as characterizations of the proteome and metabolome. In this data article, we describe the methods used to produce the data and present the raw sequence read data in FASTQ files, sequencing run statistics and a summary flatfile of raw counts for 22,164 genes across 31 samples, representing 3-5 biological replicates at each timepoint. We propose that this data will be a valuable contribution to diverse research efforts in bioinformatics, stem cell research and developmental neuroscience studies.

## Objectives

Transcriptional profiling by RNA sequencing (RNAseq) enables the sensitive and accurate characterization of the transcriptome ^1– 4^. Following enumeration of reads, various methods are available to normalize counts, estimate probability distributions and identify differential gene expression between biological conditions ^3, 5, 6^. RNAseq has the added advantages of being extremely high-throughput and relatively inexpensive, with a high signal-to-noise ratio and a dynamic range encompassing 4–5 orders of magnitude. This combination of throughput and sensitivity enables the detection of rare transcripts from nanograms of RNA.

We have described a method to produce large quantities of highly enriched, electrically active glutamatergic neurons (ESNs) from suspension-adapted mouse embryonic stem cells (ESCs) ^7, 8^. This technique is a modification of the 4/4 method, and is based on the spatiotemporal changes in morphogens that occur during neural induction and patterning ^9– 11^. In this method, neuroepithelial stem cells (NESCs) are derived from ESCs by the withdrawal of leukemia inhibitory factor (LIF), then induced to undergo neurogenesis and neural patterning by supplementation with all- *trans* retinoic acid (RA) in the presence of fetal bovine serum ^12^. We have modified the 4/4 method to include feeder cell-free, suspension culture of ESNs; differentiation under rotary conditions to normalize the intra-aggregate environment; and neuronal induction and neural induction and patterning using 6 µM RA. These refinements have resulted in a facile and economical method to generate large quantities of highly enriched glutamatergic neurons ^13^. Using immunocytochemistry, we have shown that ESNs are composed mostly of glutamatergic neurons (~95%), with about 5% GABAergic neurons, and no evidence of dopaminergic, serotonergic, cholinergic or glycinergic subtypes ^7^. Expression profiling using RNA sequencing has confirmed that derived cultures are primarily glutamatergic, and identified the abundant expression of a wide range of cortical markers, including reelin, Pax6, Otx1, Ctip2 and Cux1/2 ^13– 19^.

Although aspects of corticogenesis have been replicated *in vitro* by the directed differentiation of pluripotent stem cells, spatiotemporal changes in gene expression responsible for regionalization and apical-to-basal patterning of the cerebral cortex are not well understood ^20– 22^. The embryological origin of the cerebral cortex is the telencephalon, which is the most anterior structure of the mammalian neural tube ^23^. Cortical glutamatergic neurons are generated by proliferation and laminar patterning of the dorsal telencephalon, whereas inhibitory GABAergic neurons derive from the ventral telencephalon and migrate tangentially into cortical layers ^24, 25^. Our ability to derive predominantly glutamatergic neurons that express markers of the telencephalon and all six cortical layers is consistent with an *in vitro* model of the developing telencephalon and cortical layer formation ^23^. We propose to apply this model to identify the temporal changes in gene and isoform expression associated with neural patterning and corticogenesis. If successful, we intend to use ESNs to functionally interrogate the roles of individual genes in executing the transcriptional programs underlying the formation of the cerebral cortex.

To evaluate the transcriptional changes associated with differentiation of cortical glutamatergic neurons, we conducted a longitudinal expression profile of the deep transcriptome during neurogenesis from days *in vitro* (DIV) -8 to 28, where DIV 0 corresponds to the end of differentiation. High quality RNA was isolated from ESCs (DIV -8; n=4); neuroepithelial stem cells (DIV -4; n=3); radial glia (DIV 0; n=3); developmental stage (DS) I/II neurons (DIV 1; n=4); DS III/IV neurons (DIV 7; n=5); and maturing DS IV/V neurons at DIV 16 (n=4); DIV 21 (n=4); and DIV 28 (n=4). The summary data, including quality scores, are presented in Data File 1, and the raw transcript read counts for each biological replicate are presented in Data File 2. The FASTQ files generated for each biological replicate are available at the Sequence Read Archive (SRA), a freely accessible database provided by NCBI ( http://www.ncbi.nlm.nih.gov/sra/), under the accession number PRJNA185305.

In addition to providing the basis for a study to characterize transcriptional processes involved in corticogenesis and neuronal maturation, we can foresee several other applications for this data. First, the identification of genes and isoforms that exhibit differential expression in synchronization with known markers is expected to provide novel insight into molecular mechanisms of neurogenesis and neural patterning. Second, a few algorithms are available for statistical determination of differential gene expression within longitudinal data sets. The depth and quality of these data suggests it may be well-suited for the development and validation of such methods. Third, we envision this dataset facilitating inter-specific comparisons of transcription during neurogenesis and corticogenesis, providing insight into transcriptional mechanisms common to and unique in the evolution of the mammalian cerebral cortex. For these reasons, we are making this data publically available for research efforts in bioinformatics, stem cell research and developmental neuroscience studies.

## Materials and methods

### Suspension adaptation and continuous culture of mouse ESCs

ESCs were adapted to feeder cell-free, suspension culture and maintained as previously described ^7, 13^. In brief, aliquots of R1 ESCs (ATCC, Manassas, VA) were thawed and maintained at 37°C at 5% CO _2_ in 90% relative humidity in 10 cm bacterial plates in ESM (Knockout DMEM supplemented with 100 µM β-mercaptoethanol, 0.1mM nonessential amino acids, 2.0 mM L-glutamine, 5000 units/mL penicillin/streptomycin, 1000 units/mL recombinant mouse LIF [all Life Technologies, Carlsbad, CA] and 15% ES qualified fetal calf serum [ATCC, Manassas, VA]) ^26^. Cells were passaged once aggregates first became clearly visible to the naked eye (4–8 days) and maintained for at least 5 passages prior to differentiation. For passaging, aggregates were allowed to settle by gravity, washed once with 0.5 mL PBS and dissociated for 3 min at 37°C with 0.5 ml of TrypLE Express (Life Technologies). Dissociation was terminated by addition of 0.5 mL ESM followed by gently trituration with a P1000 pipette to achieve a single-cell suspension. ESNs were counted manually using a hemocytometer and ~1.5×10 ^6^ mESCs were transferred to 10 mL ESM in a fresh 10 cm bacterial dish.

### Neuronal differentiation

ESCs were differentiated into neurons between 5–30 passages after adaptation to suspension culture. A modified 4/4 protocol was used for neuron differentiation ^7, 27^. Following routine sub-passaging, 3.5×10 ^6^ mouse ESCs were transferred to 30 mL differentiation medium (ESM modified to contain 10% ESC-qualified fetal calf serum and without LIF) in a 10 cm ultra-low attachment suspension culture dish (Corning, Lowell, MA). This was designated as DIV -8. Differentiating aggregates were maintained on a rotary shaker at 45 rpm at 37°C, 5% CO2 and 90% relative humidity. Complete media changes were conducted every 48 h, and media was supplemented with 6 µM retinoic acid (Sigma-Aldrich) at DIV -4 and DIV -2.

On DIV 0, aggregates were dissociated with TrypLE Express for 5 min at 37°C. Trypsinization was halted with 5 mL of 1% soybean trypsin inhibitor (Life Technologies), the aggregates were gently dissociated by trituration with a 10 mL pipet, and the cell suspension was filtered through a 40 µm cell strainer (Thermo Scientific, Waltham, MA). Cells were pelleted for 5 min at 300 x g, washed in N2 medium (Neurobasal-A medium with 1 x N2 vitamins, 2 mM glutamine and antibiotics [Life Technologies]) and counted manually using a hemocytometer. Neuronal progenitors were plated at 1.5×10 ^6^ cells/cm ^2^ in poly-D coated dishes. Complete washes with N2 medium were conducted at 4 h and 24 h to remove residual serum and non-adherent cells. At DIV 2, N2 was replaced with B27 medium (Neurobasal-A supplemented with antibiotics, 2 mM glutamine and 1 x B27 vitamins [Life Technologies]). Subsequently, ESNs underwent full medium changes with B27 on DIV 4, 8 and 12. On DIV 8 the media was supplemented with 30 µM 5-fluoro-2’-deoxyuridine and 70 µM Uridine (Sigma-Aldrich) to select against any remaining glia. Following DIV 12, ESNs were left undisturbed until RNA harvest. Cultures remained healthy and viable until at least DIV 28 under these conditions.

### Expression profiling

Neurons were harvested from 6 cm dishes at DIV -8, -4, 0, 1, 7, 16, 21 and 28 (n=3 to 5) and RNA was isolated by QIAcube (Qiagen, Valencia, CA) using the RNeasy mini kit protocol (Qiagen) and submitted to Expression Analysis, Inc. (Durham, NC) for library preparation and sequencing. Typical recovery was ~3 µg per 6 cm dish, with an RNA integrity number exceeding 8 and a 260:280 of 1.8 – 2.2. PolyA+ RNA was purified from 500 ng of total RNA using polyA selection, chemically fragmented and reverse transcribed using random hexamers. This was followed by second strand synthesis and end repair. Libraries were prepared for paired-end sequencing using the TruSeq™ RNA sample prep kits (Illumina, San Diego, CA) per manufacturer’s instructions, and library size and integrity were determined using the Agilent Bioanalyzer 2100 (Santa Clara, CA). Libraries were bound to flow cell surfaces using the Standard Cluster Generation Kit v5 (Illumina). Flow cells were transferred to the Illumina HiSeq 2000 and run using TruSeq SBS Kits (Illumina). Paired-end sequencing data were generated over 2x50 sequencing cycles. Sequence information and quality scores are available at SRA in FASTQ format, with the forward and reverse reads appended with an "F" or "R" (e.g., DIV7.1F and DIV7.1R). Raw sequence reads were aligned using the University of California, Santa Cruz’s mouse knownGENE track, and transcriptome abundance estimation was performed on completed alignments using RSEM (RNA-Seq by Expectation Maximization) ^28^. A summary flatfile containing the total raw reads for each gene in each biological replicate is presented in Data File 2. Genes were excluded if no reads occurred in any biological sample.

Data File 1: summary data RNA-sequencing by Expectation Maximization summary of quality metrics for paired-end Illumina runs. The last column indicates the filenames of the corresponding FASTQ files available at Sequence Read Archive, appended with an “F” for the forward sequence read or an “R” for the reverse sequence read.

Data File 2: raw transcript read counts RNA-Sequencing by Expectation Maximization-estimated total counts for all genes in biological replicates of days in vitro (DIV) -8, -4, 0, 1, 7, 16, 21 and 28 samples. Genes for which no reads were detected in any biological sample were excluded.
