# Supplementary material for: Longitudinal RNA sequencing of the deep transcriptome during neurogenesis of cortical glutamatergic neurons from murine ESCs
Source: F1000Res. 2013 Feb 7;2:35. [Version 1] doi: 10.12688/f1000research.2-35.v1 (PMC3829120; doi:10.12688/f1000research.2-35.v1)
Supplement: Peer review history

# Peer review: Referee response for version 1

**Published:** 2013-05-14

**Authors:** Joyce van de Leemput

**Affiliations:** 1 Neural Stem Cell Institute, Rensselaer, NY, USA

The depth and temporal nature of the dataset presented in this paper will be beneficial to any researcher interested in cortical development in general, and potentially lead to many new insights and avenues to pursue. 

A point of note, in my experience differences in passage number of the cells used for differentiation can affect gene expression levels throughout. The authors state “ESCs were differentiated into neurons between 5-30 passages after adaptation to suspension culture.”, I wonder if that is why the DIV21 samples cluster in between the DIV16 and DIV28 when performing a PCA analysis on the transcript read counts (obtained from Data File 2)? 

Related question, how raw are the transcript read counts in Data File 2, as I thought raw counts would have to be integers whereas the counts given have decimal points? 

Finally, with regard to the previous Ref Report (Ragsdale and Albertin; 12 March 2013), have you considered comparative analysis using the Allen Brain Atlas/ Mouse Brain expression data for the thalamic and cortical areas and see which region your samples resemble most?

I have read this submission. I believe that I have an appropriate level of expertise to confirm that it is of an acceptable scientific standard.

# Peer review: Referee response for version 1

**Published:** 2013-03-12

**Authors:** Cliff Ragsdale, Caroline Albertin

**Affiliations:** 1 Department of Neurobiology, University of Chicago, Chicago, IL, USA; 2 Department of Organismal Biology and Anatomy, University of Chicago, Chicago, IL, USA

There are a growing number of protocols for differentiating stem cells into particular neural cell types. This paper demonstrates the great potential of RNAseq technologies for assessing the identities of such differentiated cells in culture. The authors’ goal is an *in vitro* population of 'glutamatergic cortical neurons'. Although many of the genes catalogued show the anticipated profiles across the differentiation process ( *Otx2* abundance decreases with DIV while *Kcnh5* reads increase), the dataset also demonstrates that this culture protocol may not be the best for 'glutamatergic cortical neuron' study as transcripts for the predominant cortical vesicular glutamate transporter gene, *Vglut1/Slc17a7*, are barely detected in the differentiated cell populations.

We have read this submission. We believe that we have an appropriate level of expertise to confirm that it is of an acceptable scientific standard.

# Response

**Published:** 2013-03-19

**Authors:** Patrick McNutt

**Affiliations:** Research Division, United States Army Medical Research Institute of Chemical Defense (USAMRICD), USA

Despite the presence of a number of transcripts suggestive of telencephalogenesis, the singular derivation of vglut2+/vglut1- neurons has bothered us as well, and was one of the motivating factors underlying this experiment. While vglut2 expression has been reported in the neocortex, transcripts have generally been described to appear postnatally (e.g., see allen brain institute expression data for slc17a6 in the developing mouse brain) and vglut1 expression is much stronger than vglut2 in cortical glutamatergic neurons. We are still wading our way through the differential gene expression data, but preliminary, analysis suggests that the RNAseq data may be more consistent with a thalamic origin than a neocortical origin.  Not only is thalamogenesis consistent with a vglut2+/vglut1- phenotype, but it is also less well described than neocortical development. We are currently working toward a clear differential diagnosis based on gene expression (should one exist) in an attempt to more clearly elucidate the structural origin of neurons derived in our model.
